# The Ethical Use of Telepsychiatry in the Covid-19 Pandemic

**DOI:** 10.3389/fpsyt.2020.00665

**Published:** 2020-07-14

**Authors:** Julia Stoll, John Z. Sadler, Manuel Trachsel

**Affiliations:** ^1^Faculty of Medicine, Institute of Biomedical Ethics and History of Medicine, University of Zurich, Zurich, Switzerland; ^2^Department of Psychiatry, The University of Texas Southwestern Medical Center, Dallas, TX, United States; ^3^Clinical Ethics Unit, University Hospital Basel and University Psychiatric Clinics Basel, Basel, Switzerland

**Keywords:** telepsychiatry, ethics, COVID 19, social justice, mental health

## Introduction

In a statement to the press, Dr. Hans Henri P. Kluge, World Health Organization (WHO) Regional Director for Europe, stressed that it is important not to lose sight of the mental health implications of Covid-19 for everyone, noting that “[i]t is absolutely natural for each of us to feel stress, anxiety, fear, and loneliness during this time” (1). If mentally healthy individuals react with fear and stress to the Covid-19 pandemic, it is not difficult to imagine that such reactions make those who have mental disorders especially vulnerable to harm.

## Challenges for Patients With Mental Disorders in Times of Covid-19

Yao, Chen, and Xu (2) listed four reasons to explain why patients with mental disorders may be at particular risk in the Covid-19 pandemic. First, patients with mental disorders may be at increased risk of infection because they are less aware of the dangers or because they adhere less to official measures. Second, poor symptom recognition combined with stigmatization means that Covid-19 infection may not be detected as quickly, and treatment following infection may be compromised by various psychiatric comorbidities. Third, patients with mental disorders may be more vulnerable to the public panic and anxiety triggered by the pandemic, which may aggravate the symptoms of the underlying mental disease. Finally, face-to-face outpatient treatment may be impeded as a result of various government measures, including quarantine. Importantly, the undersupply of services for patients with mental disorders not only increases existing healthcare inequities but potentially facilitates the spread of Covid-19 through increased infection and difficulties with adherence with public health restrictions among these patients (2). How can continuous care for patients with mental disorders be guaranteed within the confines of social distancing?

## Telepsychiatry as a Possible Solution

Telepsychiatry has been discussed as a possible solution for the care of patients with mental disorders (3, 4) and is increasingly used worldwide during the Covid-19 pandemic (5). Telepsychiatry is already well established in some countries like Australia and Canada and the effectiveness of telepsychiatry, as well as the satisfaction of its users, has been shown in various studies (6–8).

Especially in a crisis like the Covid-19 pandemic, the treatment of patients through telepsychiatry shows some specific advantages: Because telepsychiatry services maintain social distance, they eliminate the risk of infection for both patients and therapists; the patient can remain at home, and the therapist can work from their home, office, or practice. In this way, psychotherapy can be maintained or initiated even under quarantine. Patients who would otherwise attend for outpatient psychotherapy can continue their treatment remotely with the same therapist, so ensuring continuity of care and potentially improving compliance and adherence. Telepsychiatry makes even brief crisis intervention possible when physical distance prevents inpatient treatment, so potentially reducing the number of hospitalizations during the Covid-19 pandemic.

## Ethical Challenges and How to Potentially Face Them

We have identified six areas of ethical challenges in delivering telepsychiatry/psychotherapy: (1) data security, privacy, and confidentiality; (2) clinical safety of telepsychiatry recipients; (3) competency and preparedness for telepsychiatric clinicians; (4) legal, regulatory, and financial concerns; (5) informed consent for services; and (6) social justice concerns. At first sight, the benefits of telepsychiatry solutions, which are relatively cost-effective and technologically straightforward, seem convincing. However, the associated ethical challenges must not be overlooked. In particular, risks to data security, privacy, and confidentiality may increase when using freely available software, which may be less secure (9). Lustgarten and colleagues (10) offer some important recommendations regarding good and safe use of technologies. Crucially, clinician should ensure that telepsychotherapy is appropriate for the patient in question. For example, this approach may not be suitable for patients with concrete suicidal ideation because rapid reaction to emergency situations may be hindered by physical distance. Kocsis and Yellowlees (11) assert a consensus around applicability for most patients with any mental disorder being treatable telepsychiatrically, unless acutely dangerous to others or themselves. Clinicians providing telepsychiatric treatment should monitor their patients for safety and refer to “actual” services accordingly. As a further prerequisite, the therapist must be qualified to provide telepsychiatric services. Implementing treatment at a distance requires particular competencies and special knowledge, which, ideally, should be trained, proven, and accredited to further assure standard of clinical care (12). Especially regarding newly emerging technologies such as the use of mobile apps, new competencies and challenges may be faced by clinicians (13). Creating and maintaining a therapeutic relationship in the virtual environment even with a previously-established therapeutic alliance may still give rise to misunderstandings, frustrations, or interruptions due to technological or network failures. Some standard psychotherapy parameters may be compromised: for example, establishing the basic standard of eye contact in the clinician-patient relationship is challenging with virtual environments. In such circumstances, the patient’s sense of familiarity or intimacy may change, especially when communicating with a therapist from the privacy of their homes (14). However, online telepsychiatric care may also offer some advantages for the therapeutic relationship, which can vary depending on the patient’s disorder (11, 13). Clinicians should explore capitalizing on these advantages. There are also potential legal implications; for example, licensing, care reimbursement, and malpractice insurance issues may arise if telepsychiatry is provided across national borders (15, 16). During the Covid-19 pandemic, some states in the USA changed their licensing requirements, sometimes allowing practitioners to treat patients even from a state where they were not licensed (5). Clinicians who practice telepsychiatry should inform themselves about the current requirements of their practice jurisdiction and where their patients are located. To allow the patient to make a careful decision about the advantages and disadvantages of telepsychiatry, clinicians should permit adequate time to provide an appropriate informed consent procedure (17). Murphy and Pomerantz (18) have compiled an updated version of a questionnaire with the most important aspects of informed consent for online psychotherapy, which should aid psychotherapists’ informed-consent practices. Still another concern regarding telepsychiatry is the social justice issue of fair access to this technology. Many low and medium-income, and even some high-income countries, have limited access to telepsychiatry and telepsychotherapy: online access, video/audio connectivity, broadband capability, a safe and private setting to engage care (which is lacking with the homeless), not to overlook access to clinicians (19). Many patients or clients may not have the technology and personnel available to them to meet our aforementioned guidance about competence and quality of services. Additionally, telepsychiatry and related services may or may not be paid by public funding. These limitations are particularly acute with the patients with sever mental disorders, whose political voice has always been marginal (20). In these situations, taking care of patients to the best of our ability should be a prevailing value. Moreover, health care inequities are not limited to any country’s income level. For example, the U.S. American Psychiatric Association has advocated for equitable payment for telephony-based, audio-only telepsychiatry to reduce inequities in access to telepsychiatry for those who do not have access to broadband cellular or wifi connectivity, which enable video-based telepsychiatry (21). We would encourage clinicians using and refining telepsychiatric services to advocate for more equitable support for such services in their respective countries.

For further ethical arguments for and against different forms of online psychotherapy, see Stoll, Müller, and Trachsel’s review (22). Concerning general guidelines, Sansom-Daly and colleagues (16) discuss some international guidelines concerning telemental health using videoconferencing. In addition, we recommend to consult the following guidelines from the APA Joint Task Force for the Development of Telepsychology Guidelines (23) and from Yellowlees and colleagues (24).

## Discussion

In conclusion, extraordinary times require extraordinary public measures and modes of treatment, and telepsychiatry may offer a means of preventing service undersupply for patients with a mental disorder during public health crises such as the Covid-19 pandemic. However, any therapist considering this approach should take due account of the individual patient’s needs and specific situation, allowing sufficient time to complete the necessary preliminaries and ensuring that they are well informed about current guidelines in the jurisdiction or state in question. Mental healthcare practitioners should always be guided by the principle that a high standard of care must be maintained in responding to the current crisis and the associated risks for the individuals with mental disorders, who are especially vulnerable to service undersupply.

Finally Shore and colleagues (5) open up some important questions: what will happen after the pandemic? Will the changes now being made by various governments, psychiatric institutions, and psychotherapists regarding telepsychiatry be maintained? How will this change and shape the professional field of psychiatry? How these questions will be answered in the future will be determined not only by health policy, but also by each individual practicing psychotherapist, their systems of practice, and also by researchers studying the topic of telepsychiatry.

## Author Contributions

JS, JZS, and MT were all involved in the writing and editing of the manuscript. All authors contributed to the article and approved the submitted version.

## Conflict of Interest

The authors declare that the research was conducted in the absence of any commercial or financial relationships that could be construed as a potential conflict of interest.

## References

[1] Kluge HHP Statement—Physical and mental health key to resilience during COVID-19 pandemic. Copenhagen, Denmark: WHO (World Health Organization) (2020). [updated 26 March 2020] (http://www.euro.who.int/en/media-centre/sections/statements/2020/statement-physical-and-mental-health-key-to-resilience-during-covid-19-pandemic).

[2] Yao H Chen J-H Xu Y-F Patients with mental health disorders in the COVID-19 epidemic. Lancet Psychiatry (2020) 7:e21. 10.1016/S2215-0366(20)30090-0 32199510 PMC7269717

[3] Torous J Myrick KJ Rauseo-Ricupero N Firth J Digital mental health and COVID-19: Using technology today to accelerate the curve on access and quality tomorrow. JMIR Ment Health (2020) 7(3):e18848. 10.2196/18848 32213476 PMC7101061

[4] Zhou X Snoswell CL Harding LE Bambling M Edirippulige S Bai X The role of telehealth in reducing the mental health burden from COVID-19. Telemed J E Health (2020) 26(4):1–3. 10.1089/tmj.2020.0068 32202977

[5] Shore JH Schneck CD Mishkind MC Telepsychiatry and the Coronavirus disease 2019 pandemic: Current and future outcomes of the rapid virtualization of psychiatric care. JAMA Psychiatry (2020) e2. 10.1001/jamapsychiatry.2020.1643 32391861

[6] Bashshur RL Shannon GW Bashshur N Yellowlees PM The empirical evidence for telemedicine interventions in mental disorders. Telemed J E Health (2016) 22(2):87–113. 10.1089/tmj.2015.0206 26624248 PMC4744872

[7] Simpson J Doze S Urness D Hailey D Jacobs P Evaluation of a routine telepsychiatry service. J Telemed Telecare (2001) 7:90–8. 10.1258/1357633011936219 11331046

[8] Hawker F Kavanagh S Yellowlees PM Kalucy RS Telepsychiatry in South Australia. J Telemed Telecare (1998) 4(4):187–94. 10.1258/1357633981932181 10505352

[9] Gamble N Boyle C Morris ZA Ethical practice in telepsychology. Aust Psychol (2015) 50(4):292–8. 10.1111/ap.12133

[10] Lustgarten SD Garrison YL Sinnard MT Flynn AWP Digital privacy in mental healthcare: current issues and recommendations for technology use. Curr Opin Psychol (2020) 36:25–31. 10.1016/j.copsyc.2020.03.012 32361651 PMC7195295

[11] Kocsis BJ Yellowlees P Telepsychotherapy and the Therapeutic Relationship: Principles, Advantages, and Case Examples. Telemed J E Health (2018) 24(5):329–34. 10.1089/tmj.2017.0088 28836902

[12] Colbow AJ Looking to the future: Integrating telemental health therapy into psychologist training. Train Educ Prof Psychol (2013) 7(3):155–65. 10.1037/a0033454

[13] Shore JH Managing virtual hybrid psychiatrist-patient relationships in a digital world. JAMA Psychiatry (2020) 77(5):541–2. 10.1001/jamapsychiatry.2020.0139 32159756

[14] Drum KB Littleton HL Therapeutic boundaries in telepsychology: Unique issues and best practice recommendations. Prof Psychol Res Pr (2014) 45(5):309–15. 10.1037/a0036127 PMC4234043 25414540

[15] Shore JH Telepsychiatry: Videoconferencing in the delivery of psychiatric care. Am J Psychiatry (2013) 170(3):256–62. 10.1176/appi.ajp.2012.12081064 23450286

[16] Sansom-Daly UM Wakefield CE McGill BC Wilson HL Patterson P Consensus among international ethical guidelines for the provision of videoconferencing-based mental health treatments. JMIR Ment Health (2016) 3(2):e17. 10.2196/mental.5481 27192931 PMC4889868

[17] Sabin JE Skimming K A framework of ethics for telepsychiatry practice. Int Rev Psychiatry (2015) 27(6):490–5. 10.3109/09540261.2015.1094034 26493214

[18] Murphy JM Pomerantz AM Informed consent: An adaptable question format for telepsychology. Prof Psychol Res Pr (2016) 47(5):330–9. 10.1037/pro0000098

[19] Fernando BL Sumathipala A Ethics of public mental health in developing societies. In: Sadler JZ Fulford KWM Van Staden W, editors. The Oxford Handbook of psychiatric ethics. Oxford: Oxford University Press (2015).

[20] Chan S Parish M Yellowlees P Telepsychiatry Today. Curr Psychiatry Rep (2015) 17(11):89–98. 10.1007/s11920-015-0630-9 26384338

[21] American Psychiatric Association (Update 5/1) Telepsychiatry and COVID-19. Washington DC: American Psychiatric Association (2020). [updated 01 May 2020] (https://www.psychiatry.org/psychiatrists/practice/telepsychiatry/blog/apa-resources-on-telepsychiatry-and-covid-19).

[22] Stoll J Müller JA Trachsel M Ethical issues in online psychotherapy: A narrative review. Front Psychiatry (2020) 10:993. 10.3389/fpsyt.2019.00993 32116819 PMC7026245

[23] Joint Task Force for the Development of Telepsychology Guidelines for Psychologists Guidelines for the practice of telepsychology. Am Psychol (2013) 68(9):791–800. 10.1037/a0035001 24341643

[24] Yellowlees P Shore J Roberts L Practice guidelines for videoconferencing-based telemental health - October 2009. Telemed J E Health (2010) 16(10):1074–89. 10.1089/tmj.2010.0148 21186991

